# Benign paroxysmal positional vertigo in a patient with persistent hypoglossal artery and bilateral madelung deformity

**DOI:** 10.1016/j.ibneur.2022.12.008

**Published:** 2022-12-20

**Authors:** Jacob M. Hands, Shaweta Khosa, William M. Rienas, Shri K. Mishra

**Affiliations:** a Olive View-UCLA Medical Center, USA; b The George Washington School of Medicine & Health Sciences, USA; c Olive View-UCLA Medical Center, University of Southern California Clinical Professor of Medicine, 14445 Olive View Drive Suite 2c136, Sylmar CA 91342, USA

**Keywords:** Persistent hypoglossal artery, Vertigo, Shox, Mutation, Otology, Madelung deformity, Aneurysm, Hemorrhage, Stroke

## Abstract

We explore a case of Benign Paroxysmal Positional Vertigo in the context of Persistent Hypoglossal Artery (PHA) and bilateral Madelung Deformity (MD). PHA is associated with a raft of major adverse cardiovascular events. MD can result from manifold conditions such as Turner’s Syndrome and mesomelic dwarfism. In this case, the patient’s positive family history of MD across generations is suggestive of inherited mutation in the Short Stature Homeobox (SHOX) Gene. We discuss the putative impact of SHOX on the genesis of Benign Paroxysmal Positional Vertigo (BPPV) in a patient with PHA and bilateral MD.

## Highlights

• Persistent Hypoglossal Artery (PHA) is a developmental condition characterized by an inability to abolish fetal anastomoses.
• Persistent Hypoglossal Artery is associated with a raft of major adverse cardiovascular and neurovascular events.
• Mutations in SHOX may be causal in the pathogenesis of BPPV in patients with inherited Madelung Deformity.
• Short Stature Homeobox genes are hypothesized to participated in the genesis of the bony labyrinth.

## Objective

To explore a case of Benign Paroxysmal Positional Vertigo (BPPV) in a patient with persistent hypoglossal artery (PHA) and bilateral Madelung (MD) deformity.

## Background

Persistent hypoglossal artery (PHA) is a rare congenital carotid-basilar anastomosis (Avcu et al., 2009, Coulier, 2018, MR, 2016, Fantini et al., 1994, Jin et al., 2018). While persistent trigeminal artery (PTA) is the most common carotid-vertebrobasilar anastomoses with prevalence estimated to be 0.1–0.2%, PHA is the second most common with reported prevalence of 0.03–0.09% PHA has been associated with various cardiovascular events, including embolic stroke (Avcu et al., 2009, Jin et al., 2018). Madelung deformity (MD) is another rare congenital condition characterized by impaired development of the radial epiphysis, prominent distal ulna, and restricted range of motion (Babu et al., 2019, Dickson et al., 2010, Knutsen and Goldfarb, 2014, Kozin and Zlotolow, 2015, Oliveira and Alves, 2011). MD is heritable and typically associated with a mutation in the Short-Stature Homeobox (SHOX) gene (Babu et al., 2019, Dickson et al., 2010, Knutsen and Goldfarb, 2014, Oliveira and Alves, 2011). Reported prevalence of MD is estimated to be <0.03% (Dickson et al., 2010).

## Case presentation

A 76-year-old female with a prior medical history of treated latent tuberculosis, diabetes mellitus, intracranial hemorrhage (ICH), osteoporosis, carotid intima media thickness (CIMT) results consistent with atherosclerosis, and right middle cerebral artery aneurysm status post-clipping presented to the emergency department complaining of new-onset vertigo for past three days. Upon presentation, temperature was 36.8 C, heart rate was 54 beats per minute (BPM), respiration rate (RR) was 15, and blood pressure (BP) was 124/74 with a PO2 of 97%.

The patient described the symptoms as feeling as though the ceiling was spinning and that she “was falling into an abyss.” The symptoms worsened when supine and resolved upon upright seating. No nausea, flushing, or loss of consciousness was associated with these events. Furthermore, the patient denied having chest pain, nor shortness of breath associated with these episodes. Upon physical examination, the patient was noted to have bilateral Madelung deformities. The patient was evaluated for suspected vertigo.

Holter monitoring (outpatient), transthoracic echocardiogram (TTE), and carotid ultrasound were performed, each of which was unremarkable. The patient noted that she had experienced similar episodes the year prior in which dizzy spells were accompanied by a “ball of fire” in her head. These episodes also abated upon seating and resolved spontaneously within several minutes.

Motor examination revealed normal findings. Cranial nerve examination was largely unremarkable, though pronounced bilateral hearing loss to high and low frequencies was documented. After further examination, the patient was assessed as having severe vertigo with right beating nystagmus induced upon performing the Dix-Hallpike maneuver to the right. Due to the presence of horizontal nystagmus, the patient was assessed as having horizontal canal BPPV specifically (​Kim et al., 2022, Kim et al., 2021; Bhattacharyya et al., 2017). The patient was unable to tolerate subsequent repositioning.

A CT scan of the head showed a right-sided craniotomy for clip ligation of an aneurysm at the right middle cerebral artery bifurcation. Further evaluation by CT angiogram of the head and neck showed a persistent right hypoglossal artery (PHA) originating from the external carotid artery feeding the basilar artery. MRI could not be performed due to the history of aneurysmal clip. Additionally, a CT of the patient's brain shows encephalomalacia in the right anterior temporal lobe and right superior temporal gyrus as well as bilateral cerebellar infarcts.

The patient later revealed that there existed a similar wrist abnormality in her mother, suggestive of an autosomal dominant short stature homeobox (SHOX) genetic disorder. Given the symptoms and initial work-up, including imaging findings, presentation was consistent with a diagnosis of BPPV. She was prescribed meclizine and an Epley maneuver worksheet and reported improvement in symptoms on a follow-up visit one month post consultation. Upon follow up 6 weeks later, the patient was well in appearance and vitals were unremarkable.

## Discussion

We described the case of a 67-year-old female with PHA and bilateral MD presenting to the ED with a diagnosis of BPPV.

PHA arises during the early stages of embryonic development (Avcu et al., 2009, Coulier, 2018, MR, 2016, Fantini et al., 1994). PHA represents an off-branching from the internal carotid artery, situated at C1-C3 (Avcu et al., 2009, Coulier, 2018, MR, 2016, Fantini et al., 1994). Pre-segmental arteries form a network of anastomoses that play an integral role in neurological maturation in the form of vascular accommodation for the developing fetal brain (Coulier, 2018, MR, 2016). In particular, the forebrain and hindbrain serve as beneficiaries of this vascular network (Coulier, 2018, MR, 2016). After 7–10 days, such anastomoses are typically obliterated. PHA has been associated with a spate of cardiovascular events including intracranial aneurysm, carotid and vertebrobasilar infarcts, intracerebral hemorrhages, as well as embolic stroke, specifically in patients with established atherosclerotic disease (Avcu et al., 2009, Coulier, 2018, Jin et al., 2018).

Bilateral MD is a rare congenital anomaly affecting the radial epiphysis, producing a prominent distal ulna and often resulting in restricted range of motion and subjective reports of discomfort (Babu et al., 2019, Dickson et al., 2010, Knutsen and Goldfarb, 2014, Kozin and Zlotolow, 2015, Oliveira and Alves, 2011). Resolution of symptoms can often be achieved by release of Vickers ligament (Knutsen and Goldfarb, 2014, Kozin and Zlotolow, 2015). Current estimates suggest that 50–66% of MD cases are bilateral and affect women in 4:1 ratio (Babu et al., 2019, Dickson et al., 2010). MD can be heritable, with mutations in the SHOX gene being reported in lineages characterized by outsized incidence of MD (Babu et al., 2019, Dickson et al., 2010). It should be noted that other conditions and pathologies may produce MD-like features which must be ruled out before the diagnosis of MD can be made (Babu et al., 2019, Kozin and Zlotolow, 2015, Oliveira and Alves, 2011). MD-like deformities can occur in the context of injury, malignancy, and pathologic mimics such as mesomelic dwarfism and aneuploidies such as Turner’s syndrome (Dickson et al., 2010, Kozin and Zlotolow, 2015, Oliveira and Alves, 2011).

While MD is present in early adolescence and tends to be apparent upon physical examination, the diagnosis of PHA is typically incidental. In this particular patient, the diagnosis of PHA was an artifact of CT. Its appearance likely bears no relation to the patient’s BPPV, though the presence of trans-generational MD may be of greater significance.

The short stature homeobox gene (SHOX), found in pseudoautomosmal region 1 (PAR1) of sex chromosomes, is highly conserved and is associated with a number of developmental conditions linked to abnormalities in hearing, skeletal development, and chromosomal expression, particularly in the context of Turner’s Syndrome (Babu et al., 2019, Dickson et al., 2010, Knutsen and Goldfarb, 2014, Oliveira and Alves, 2011). SHOX is involved in dyschondrosteosis, and its expression is evident in osteogenic lines, especially those of developing limbs (Dickson et al., 2010, Oliveira and Alves, 2011). Complete loss of function (LoF) SHOX mutation has been classically associated with severe skeletal abnormalities such as Langer Mesomelic Dysplasia, while insufficient expression has been tied to a broad range of conditions including but not limited to idiopathic short stature, Turner’s syndrome, and Leri-Weil dyschondrosteosis (Gürsoy et al., 2020). SHOX expression has also been confirmed in cardiac and skeletal muscle cells (Oliveira and Alves, 2011). Perhaps most intriguing is the finding that SHOX has been implicated in the development of skeletal elements of the external and middle ear (Oliveira and Alves, 2011, Gürsoy et al., 2020). Indeed, in multiple case reports detailing patients, including a cohort of 5 siblings, with total and partial SHOX LoF, disturbances in audiologic dysfunction was noted, namely conductive hearing loss, in combination with prominent abnormalities in the development of middle ear structures (Gürsoy et al., 2020, Nassif and Harboyan, 1970, De Leenheer et al., 2003, Lucchetti et al., 2018, Delil et al., 2016). That SHOX may be directly involved in hearing disturbance via impaired development of the ear’s bony structures cannot be disregarded. Though BPPV may be described as idiopathic, we argue that the incidence of BPPV in subjects with MD may require further examination, especially as it pertains to pathogenic mutations within SHOX.

## Conclusion

Our patient had an inherited congenital Madelung deformity suggestive of a SHOX gene disorder along with PHA in the setting of BPPV. The relationship between MD and BPPV owing to SHOX mutation cannot be disregarded. Future studies may be warranted to further examine this anecdotal finding.

## CRediT authorship contribution statement

Jacob M. Hands (JMH), Shaweta Khosa (SK), William M. Rienas (WMR), and Shri K. Mishra (SKM), conceptualized and ideated this piece collectively. JMH wrote and edited this manuscript with assistance from SK, WMR and SKM. SK and SKM supervised the creation of this work and guided its development. SKM is the senior author and clinical supervisor.

## Funding disclosure

No outside sources of funding were used in the preparation of this manuscript.

## References

[bib1] Avcu S., van der Schaaf I., Ozcan H.N., Sengul I., Fransen H. (2009). Persistent hypoglossal artery detected incidentally in a hypertensive patient with intracerebral hemorrhage: a case report and review of the literature. Cases J..

[bib2] Babu S., Turner J., Seewoonarain S., Chougule S. (2019). Madelung’s deformity of the wrist—current concepts and future directions. J. Wrist Surg..

[bib3] Bhattacharyya N., Gubbels S.P., Schwartz S.R. (2017). Clinical practice guideline: benign paroxysmal positional vertigo (Update). Otolaryngol. Neck Surg..

[bib4] Coulier B. (2018). Persistent hypoglossal artery. J. Belg. Soc. Radiol..

[bib5] De Leenheer E.M., Oudesluijs G.G., Kuijpers-Jagtman A.M., Rappold G.A., Sengers R.C., Cremers C.W. (2003). Congenital conductive hearing loss in dyschondrosteosis. Ann. Otol. Rhinol. Laryngol..

[bib6] Delil K., Karabulut H.G., Hacıhamdioğlu B. (2016). Investigation of SHOX gene mutations in Turkish patients with idiopathic short stature. J. Clin. Res Pedia Endocrinol..

[bib7] Dickson J., Williams D., Standley D. (2010). Traumatic injury to a wrist with incidental Madelung’s deformity. Orthop. Traumatol.: Surg. Res..

[bib8] Fantini G.A., Reilly L.M., Stoney R.J. (1994). Persistent hypoglossal artery: diagnostic and therapeutic considerations concerning carotid thromboendarterectomy. J. Vasc. Surg..

[bib9] Gürsoy S., Hazan F., Aykut A. (2020). Detection of *SHOX* gene variations in patients with skeletal abnormalities with or without short stature. J. Clin. Res Pedia Endocrinol..

[bib10] Jin X., Sun L., Feng Z., Li X., Zhang H., Meng K., Yu W., Fu C. (2018). Persistent hypoglossal artery as a potential risk factor for simultaneous carotid and vertebrobasilar infarcts. Front. Neurol..

[bib11] Kim E.K., Pasquesi L., Sharon J.D. (2022). Examining migraine as a predictor of benign paroxysmal positional vertigo onset, severity, recurrence, and associated falls. Cureus.

[bib12] Kim H.J., Park J., Kim J.S. (2021). Update on benign paroxysmal positional vertigo. J. Neurol..

[bib13] Knutsen E.J., Goldfarb C.A. (2014). Madelung’s deformity. HAND.

[bib14] Kozin S.H., Zlotolow D.A. (2015). Madelung deformity. J. Hand Surg..

[bib15] Lucchetti L., Prontera P., Mencarelli A. (2018). Report of a novel SHOX missense variant in a boy with short stature and his mother with leri-weill dyschondrosteosis. Front Endocrinol..

[bib16] MR S. (2016). Persistent Primitive Hypoglossal Artery (PPHA) – a rare anomaly with literature review. J. Clin. Diagn. Res..

[bib17] Nassif R., Harboyan G. (1970). Madelung's deformity with conductive hearing loss. Arch. Otolaryngol..

[bib18] Oliveira C.S., Alves C. (2011). The role of the SHOX gene in the pathophysiology of Turner syndrome. Endocrinol. Y. Nutr..

